# Corrosion Behavior of Homogenized and Extruded 1100 Aluminum Alloy in Acidic Salt Spray

**DOI:** 10.3390/ma17061279

**Published:** 2024-03-10

**Authors:** Yuchao Zhao, Qiang Lu, Qudong Wang, Dezhi Li, Feng Li, Yuzhao Luo

**Affiliations:** 1 National Engineering Research Center of Light Alloy Net Forming, State Key Laboratory of Metal Matrix Composites, School of Materials Science and Engineering, Shanghai Jiao Tong University, Shanghai 200240, China; zhaoyuchao@sjtu.edu.cn (Y.Z.); luqiang951127@sjtu.edu.cn (Q.L.); 2 Warwick Manufacturing Group, University of Warwick, Coventry CV4 7AL, UK; dezhi.li@warwick.ac.uk; 3 GD Midea Heating & Ventilating Equipment Co., Ltd., Foshan 528308, China; lifeng3@midea.com (F.L.); luoyz1@midea.com (Y.L.)

**Keywords:** 1100 aluminum alloy, homogenized, hot-extruded, acid salt spray, pitting corrosion

## Abstract

The 1100 aluminum alloy has been widely used in many industrial fields due to its high specific strength, fracture toughness, excellent thermal conductivity, and corrosion resistance. In this study, the corrosion behavior of the homogenized and hot-extruded 1100 aluminum alloy in acid salt spray environment for different time was studied. The microstructure of the 1100 aluminum alloy before and after corrosion was characterized by an optical microscope (OM), scanning electron microscopy (SEM), X-ray photoelectron spectroscopy (XPS), and a laser scanning confocal microscope (LSCM). The difference in corrosion resistance between the homogenized and extruded 1100 aluminum alloy was analyzed via the electrochemical method. The results indicate that after hot extrusion at 400 °C, the microstructure of the 1100 aluminum alloy changes from an equiaxed crystal structure with (111) preferentially distributed in a fibrous structure with (220) preferentially distributed. There was no obvious dynamic recrystallization occurring during extrusion, and the second-phase particles containing Al-Fe-Si were coarse and unevenly distributed. With the increase in corrosion time, corrosion pits appeared on the surface of the 1100 aluminum alloy, and a corrosion product layer was formed on the surface of the homogenized 1100 aluminum alloy, which reduced the corrosion rate. After 96 h of corrosion, the CPR of the extruded samples was 0.619 mm/a, and that of the homogenized samples was 0.442 mm/a. The corrosion resistance of the extruded 1100 aluminum alloy was affected by the microstructure and the second phase, and no protective layer of corrosion products was formed on the surface, resulting in a faster corrosion rate and deeper corrosion pits.

## 1. Introduction

The 1100 alloy is a pure aluminum alloy with high specific strength, high fracture toughness, excellent thermal conductivity, and strong chemical corrosion resistance and weathering resistance. It has become one of the most versatile, cost-effective, and attractive metal materials being widely used in different industrial fields [1,2]. With the advocacy for global energy conservation and emission reduction, research on the corrosion performance of designed aluminum parts has found that the corrosion rate of aluminum alloys under acidic condensation conditions is much lower than that of certain steel grades [3].

It is well known that the corrosion resistance of aluminum alloy depends on the formation of alumina passive films on its surface [4,5]. In the pH range of about 4.5–8, this surface film is usually stable [6]. However, most strong acids and alkalis can dissolve the oxide layer, resulting in faster corrosion of aluminum. The alumina layer is also eroded by atmospheric chlorides or sulfides. The adsorption of chloride ions (Cl^−^) in the protective film faults and the penetration and accumulation of Cl^−^ in these defects are considered to be triggering factors for the pitting nucleation process [7,8]. Therefore, it is of great significance to study the rate and mechanism of oxidation layer degradation in the presence of chloride or sulfide in highly acidic environments.

In the past, the research on the corrosion behavior of 1100 aluminum alloys was more focused on the corrosion behavior under near-neutral conditions after immersion in simulated seawater solution (3.5 wt.% NaCl). For example, S. A. Zakaria et al. [9] studied the effects of annealing and solution treatment on the mechanical properties and corrosion properties of cold-rolled Al-1100 alloys in 3.5 wt.% NaCl solution and discussed the effect of microstructure on the corrosion properties. Marlon L. Mopon et al. [7] studied the corrosion behavior of anodic aluminum oxide in simulated seawater solution by anodizing AA-1100 aluminum alloys. Hosni Ezuber et al. [10] studied the corrosion behavior of AA5083 and AA1100 aluminum alloys in seawater at 23 and 60 °C, and showed that the types of intermetallic particles in the aluminum alloy played a major role in passivity breakdown and pit morphology in seawater. Vasundhara Shinde et al. [11] studied the corrosion of Al 1100 alloys in an acidic condensation environment and found that the formation of acidic condensation droplets led to uneven corrosion of Al 1100 samples. Thus, immersion corrosion is a kind of uniform corrosion which cannot effectively characterize the corrosion behavior of metal materials in an atmospheric environment.

The salt spray test is an environmental test that uses artificially simulated salt spray environmental conditions created by salt spray test equipment to confirm the corrosion resistance of products or metal materials, and the relevant standards are ASTM G 85-2011, ISO 9227:2022, etc. [12,13]. Some articles have reported the corrosion of aluminum alloys in salt spray environments. Jing-zhen Qiao et al. [14] studied the corrosion behavior and microstructure of 2024 aluminum alloy sheets after shot peen forming in a salt spray environment. Surendarnath et al. [15] used traditional and new equal-channel angular pressing (ECAP) molds to process commercial pure aluminum, and studied the corrosion of annealed and ECAP-treated samples using the immersion test and salt spray test. However, most studies have used the neutral salt spray test, which corrodes slowly and takes a long time. In order to quickly evaluate the corrosion resistance of aluminum alloys, an acidic environment can be used to accelerate corrosion [16,17,18,19].

The purpose of this study is to study the corrosion of aluminum 1100 alloys in an acidic salt spray environment. In this study, the homogenized 1100 aluminum alloy ingots were prepared and hot-extruded. The corrosion resistances of homogenized and extruded 1100 aluminum alloys after acid salt spray for different times were compared and analyzed according to the microstructure, corrosion morphology, and an electrochemical test. It is believed that the results of this work will help to provide anti-corrosion data and a theoretical basis for the practical engineering application of 1100 aluminum alloys in acidic environments, such as in the marine engineering and chemical industries.

## 2. Materials and Methods

### 2.1. Materials

Homogenized 1100 aluminum alloy was obtained through homogenizing heat treatment of commercial 1100 aluminum alloy ingots with diameters of 178 mm. The commercial 1100 aluminum alloy ingots were provided by Henan Runze Light Alloy Technology Co., Ltd. (Pingdingshan City, China), and their composition is shown in Table 1. The homogenizing heat treatment process was as follows: the ingots were placed in a furnace (KSL-1200X-J, Sino-us Joint Venture Hefei Kejing Material Technology Co., LTD, Hefei City, China) at 595 ± 5 °C for 12 h, followed by air cooling to 250 °C and rapid cooling with strong wind until room temperature was reached. Then, the homogenized 1100 aluminum alloy ingots were hot-extruded to make a strip sample with a cross-section of 25 mm × 2 mm. The extrusion process was as follows: the homogenized 1100 aluminum alloy was held at 540 °C for 0.5 h before extrusion, the extrusion cylinder temperature was 400 °C, and the extrusion was carried out at a maximum extrusion ratio of 55:1.

### 2.2. Acid Salt Spray Test

The samples for the salt spray corrosion test were divided into 5 groups and tested for different durations, i.e., 0, 24, 48, 72, and 96 h, with 5 samples in each group. Samples were cleaned with anhydrous ethanol solution and dried before corrosion, and the initial mass of each sample was weighed using an analytical balance (XPR106DUH/AC, Mettler-Toledo, Switzerland) with a range of 120 g and a readability of 0.005 mg. The acetic acid salt spray (AASS) corrosion test was conducted in accordance with the ISO 9227:2022 standard. The exposure zone of the acetic acid–salt spray fog chamber was maintained at 35 ± 2 °C, and the temperature in the saturator tower was 47 °C. The salt solution was 50 g/L sodium chloride (NaCl), and the pH of around 3 was adjusted by a few drops of acetic acid. The block-shaped samples were tilted and placed in a V-shaped groove at a 30° angle to the vertical direction. The samples for salt spray testing could be recovered at different time intervals, up to a maximum of 96 h.

The weight loss analysis was calculated according to the difference in the weight of the samples before and after corrosion. After the acid salt spray test, the samples were ultrasonically cleaned in distilled water to remove the corrosion products in the corrosion pit before weighing.

### 2.3. Electrochemical Tests

Electrochemical tests were performed on DH3000 electrochemical workstation (Donghua Analytical Instruments Co., Ltd., Jingjiang City, China) in 50 g/L NaCl acetic acid solution (with pH = 3.0–3.1) using the conventional three-electrode system. The platinum sheet was used as the counter electrode, and the saturated calomel electrode was used as the reference electrode. The 1100 aluminum alloy samples were used as the working electrodes. The samples were immersed in electrolyte solution for at least 30 min, and then the open circuit potential (OCP) was measured until it became stable. All the other electrochemical tests were conducted based on a stable OCP.

Electrochemical impedance spectroscopy (EIS) was used to evaluate the corrosion behavior of the alloys in a frequency range from 100 kHz to 10 mHz. The potentiodynamic polarization tests were carried out in a potential range of −0.6~0.4 V vs. OCP, with a scanning rate of 1.0 mV/s. The polarization curves of the dynamic potential were measured to characterize the corrosion behavior of the samples.

### 2.4. Microstructural Characterization

An optical microscope (OM, LEICA DM 4000, Wetzlar, Germany) was used to examine the metallographic structure of the samples. Field emission scanning electron microscopy (SEM, Nova Nano SEM 230, Waltham, MA, USA) equipped with X-ray energy-dispersive spectroscopy (EDS) was used to characterize the corrosion morphology and corrosion product composition. The crystal structure of the samples was analyzed via X-ray diffraction (XRD, Rigaku UltimaIV, Tokyo, Japan). The three-dimensional morphology of the corrosion pits was characterized using a laser scanning confocal microscope (LSCM, Keyence VK-X3000, Osaka, Japan).

### 2.5. Surface Composition Analysis

The composition of corrosion products of the homogenized and extruded 1100 aluminum alloys were determined by X-ray photoelectron spectroscopy (XPS). The XPS experiments were carried out using ESCALAB Xi+ (Thermo Scientific, Waltham, MA, USA). The X-ray source was monochromatic Al Kα (1486.6 eV), with a power of 150 W and a flux of 20 eV. The signal in the vertical exit direction of the sample was collected. The C 1s peak from adventitious carbon at 284.8 eV was used as a reference to correct the charging shifts. Thermo Avantage software v5.99 was used to fit the data of the XPS experiment. In order to extract more reliable information, we recorded only core level spectra for the elements with the highest photoionization cross sections.

## 3. Results and Discussion

### 3.1. Microstructure Analysis

**Figure 1:**
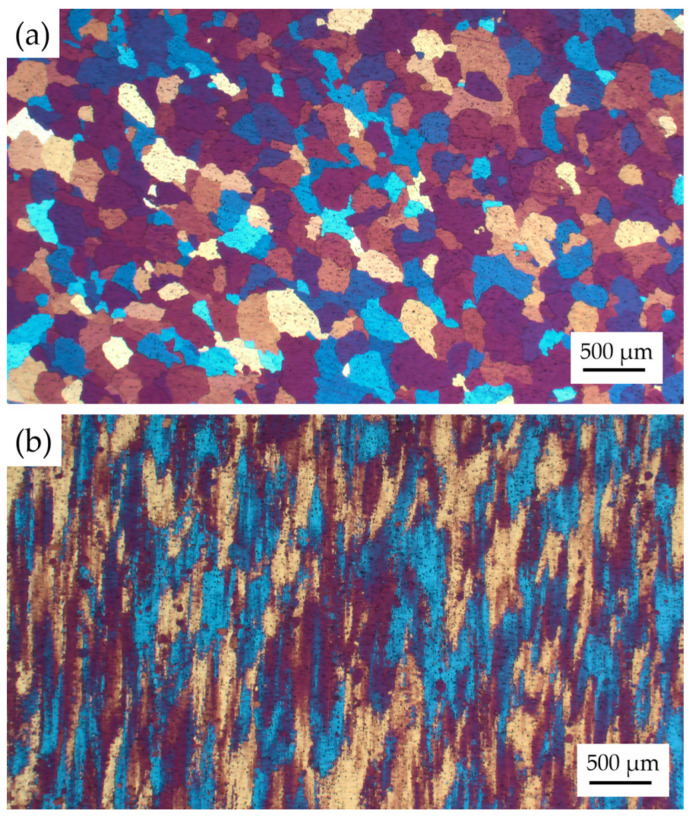
Microstructure of the homogenized (**a**) and extruded (**b**) 1100 aluminum alloy.

Prior to the corrosion test, the microstructure of the homogenized and extruded 1100 aluminum alloy was analyzed, as shown in Figure 1. The grains of the homogenized 1100 aluminum alloy were equiaxed with a grain size of about 300 μm, while the microstructure of the extruded 1100 aluminum alloy showed an elongated, fibrous structure along the extrusion direction with uneven grain size and no obvious dynamic recrystallization.

**Figure 2:**
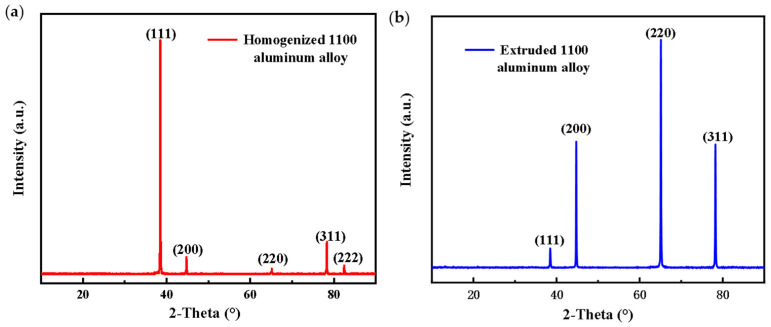
X-ray diffraction pattern of the homogenized (**a**) and extruded (**b**) 1100 aluminum alloy.

Figure 2 shows the X-ray diffraction patterns of the homogenized and extruded 1100 aluminum alloy. The results indicate that the preferred orientation of the homogenized 1100 aluminum alloy was along the plane (111), and the grain arrangement orientation changed after hot extrusion, in which the plane (220) had the highest intensity, indicating that the preferred orientation of the extruded 1100 aluminum alloy was along the plane (220).

**Figure 3:**
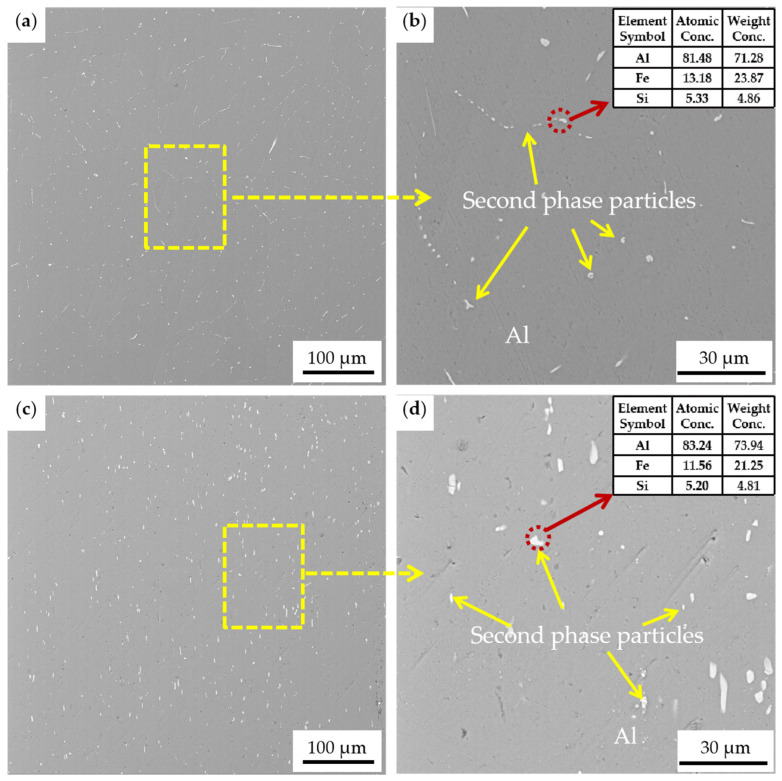
SEM images of the homogenized (**a**,**b**) and extruded (**c**,**d**) 1100 aluminum alloy; (**b**) and (**d**) are the enlarged areas of the yellow boxes in (**a**,**c**), respectively.

The 1100 aluminum alloy is composed of industrial pure aluminum, and usually contains iron and silicon as impurities. Iron is almost insoluble in aluminum and exists in the form of the intermetallic compound FeAl_3_ [20]. When silicon coexists, it exists in the form of Fe-Al-Si intermetallic compounds, such as α(Fe, Si) and β(Fe, Si) [21]. The SEM images of the homogenized (a,b) and extruded (c,d) 1100 aluminum alloy are shown in Figure 3. The microstructure of 1100 aluminum alloy after homogenization was uniform and dense, and the particles of the second phase (Al-Fe-Si) in the alloy were fine and presented a network distribution. After hot extrusion, the Al-Fe-Si phase particles in the extruded 1100 aluminum alloy were coarsened and dispersed in the aluminum matrix in a short rod-like form, with voids introduced and evenly distributed in the matrix. There is a large extrusion ratio during hot extrusion, which introduces a large number of dislocations in the aluminum matrix and some voids and defects around the second-phase particles. In the meantime, the heating from the extrusion mold (400 °C) and the heat generated during the extrusion causes the second phases to grow (coarsening). This coarse intermetallic particle feature has been reported in the literature [20], where the coarse typical composition of AA1100 alloy has been identified as containing (Al, Fe) and (Al, Fe, Si) particles. Through EDS spectrum analysis, as shown in Figure 3b,d, we found that the second phase of the 1100 aluminum alloy used in this study corresponded to Al_12_Fe_3_Si_2_, Al_6_Fe, or Al_3_Fe.

### 3.2. Rate of Mass Loss and Corrosion Penetration

**Figure 4:**
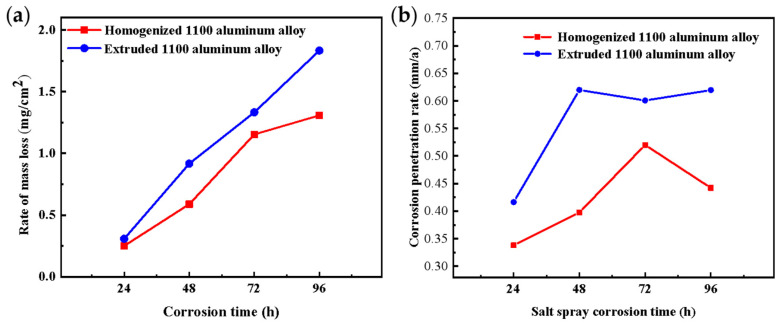
The rate of mass loss (**a**) and corrosion penetration (**b**) of the homogenized and extruded 1100 aluminum alloy for different acidic salt spray corrosion times.

After the corrosion test, weight loss analysis was carried out on the sample, and the results are shown in Figure 4a. The results of corrosion weight loss corresponded to the results of corrosion morphology, and the corrosion of the extruded 1100 aluminum alloy was more severe. After 96 h of corrosion, the weight loss rate of the extruded samples was 1.83 mg/cm^2^, and that of the homogenized samples was 1.31 mg/cm^2^. The corrosion penetration rate (CPR) of the samples was calculated according to the following equation [22]:

CPR = K*W*/*ρAt* (1)

where *W* is the weight loss of the sample after exposure time *t*; *ρ* and *A* represent the density and the area of the exposed specimen, respectively; and K is a constant whose magnitude depends on the unit area used. CPR represents the depth of material loss per unit of time, which can be conveniently expressed in millimeters per year (mm/a), where K = 87.6, and *W*, *ρ*, *A*, and *t* are expressed in milligrams (mg), grams per cubic centimeter (g/cm^3^), square centimeters (cm^2^), and hours (h), respectively.

Figure 4b shows the CPR of the homogenized and extruded 1100 aluminum alloy after different acidic salt spray corrosion times. The CPR of the extruded 1100 aluminum alloy was greater than that of the homogenized aluminum alloy. After 96 h of corrosion, the CPR of the extruded samples was 0.619 mm/a, and that of the homogenized samples was 0.442 mm/a. The CPR of the homogeneous 1100 aluminum alloy reached the maximum after 72 h, while the CPR of the extruded 1100 aluminum alloy reached the maximum after 48 h, and then the CPR decreased. This is also the reason for the greater weight loss of the extruded 1100 aluminum alloy.

### 3.3. Surface Morphology and Corrosion Products

**Figure 5:**
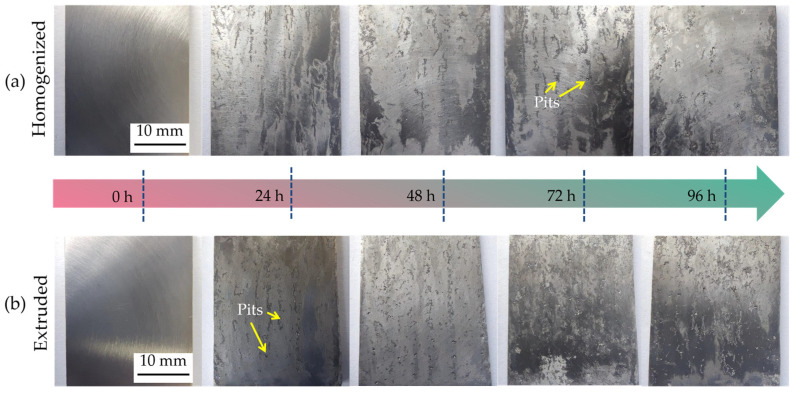
Macroscopic morphology of the homogenized (**a**) and extruded (**b**) 1100 aluminum alloys for different acidic salt spray corrosion times.

Figure 5 shows the macroscopic morphology of the homogenized and extruded 1100 aluminum alloy samples corroded by acid salt spray at different times. Aluminum alloys have a stable and tough oxide layer on the surface to protect them from corrosion, but when there are defects, such as cracks and scratches, in the oxide layer, these defects cannot be repaired due to dissolution, as pitting corrosion will happen. It can be seen from Figure 5 that the surface of the sample before salt spray corrosion was smooth, bright, and without corrosion pits. After corrosion, the surface color of the sample changed obviously, losing its smooth and shining appearance, due to the growth of corrosion pits and the deposition of corrosion products. With the increase in corrosion time, the number, depth, and diameter of pits gradually increased, and various corrosion pits began to connect with each other. After being placed in the salt spray tests for 24 h, there was no obvious pitting point on the surface of the homogeneous 1100 aluminum alloy, but there were obvious small corrosion pits on the surface of the extruded 1100 aluminum alloy. In addition, under the same corrosion time, there were more pits on the surfaces of the extruded 1100 aluminum alloy samples, which were deeper and larger. This indicates that the extruded structure is more prone to corrosion, and the corrosion rate is faster than the homogeneous 1100 aluminum alloy.

**Figure 6:**
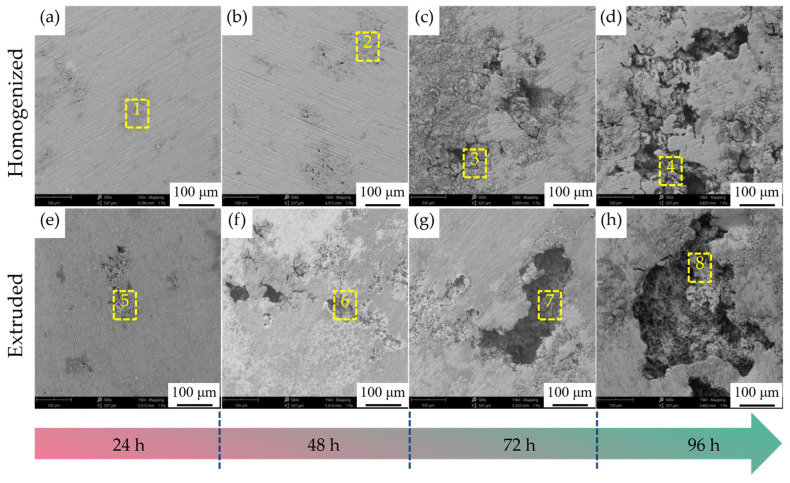
SEM images of the homogenized (**a**–**d**) and extruded (**e**–**h**) 1100 aluminum alloys for different acidic salt spray corrosion times (24 h, 48 h, 72 h, 96 h).The yellow area marked 1–8 in (**a**–**h**) indicates the EDS test area.

In order to further analyze the corrosion situation and corrosion products of the specimen, SEM was used to observe the corrosion surface of the homogenized and extruded 1100 aluminum alloy, as shown in Figure 6. It can be seen that, with the increase in the corrosion time, the number of corrosion pits formed on the surface and the size and depth of the corrosion pits increased. At the same corrosion time, the corrosion pits of the extruded 1100 aluminum alloy were larger and deeper than that of the homogeneous 1100 aluminum alloy. In the homogeneous 1100 aluminum alloy, no obvious deep corrosion pit was formed within 48 h of corrosion, but the corrosion pits showed significant growth at 72 h. Moreover, pitting preferentially occurred around the second-phase particles; it spread along the direction of the distribution of the second phase, indicating that the corrosion rate of the homogeneous 1100 aluminum alloy was first slow and then accelerated.

The corrosion layers of the homogenized and extruded 1100 aluminum alloys and the products in the corrosion pits were analyzed by energy spectrum analysis (EDS), and the results are shown in Table 2. According to the results of the energy spectrum analysis, it can be seen that in the initial stage of corrosion, the surface corrosion layers of 1100 aluminum alloys mainly consisted of Al_2_O_3_ and Al(OH)_3_. The corrosion product in the corrosion pits after 96 h was a mixture of Al_2_O_3_, Al(OH)_3_, and AlCl_3_, as well as a possible intermediate substance, Al(OH)Cl_2_ [9,16]. There is naturally a stable and tough aluminum oxide film on the surfaces of aluminum alloys to protect the alloy from corrosion, but this oxide film is only stable at pH values between 4 and 8 [8,23], below and above this range, the oxide film can be locally dissolved. The AASS corrosion solution contains a large number of Cl^−^, which can be absorbed on the oxide film, and when the Cl^−^ reach a certain concentration, it gathers together and destroys the oxide film in the acidic environment, allowing the salt spray solution to contact and corrode the aluminum alloy matrix. This is a typical pitting corrosion that creates pits on the surface of an aluminum alloy. Dissolved Al^3+^ does not diffuse easily. As Al^3+^ increases in the pits, Cl^−^ enters the mine to maintain electrical neutrality. At this point, the corrosion pits form an AlCl_3_ solution, thus maintaining the activity of the aluminum alloy surface. In addition, the acidity of the solution increases due to the hydrolysis of Cl^−^ and Al^3+^ ions. The increase in acidity in the pit accelerates the dissolution rate of the anode.

**Figure 7:**
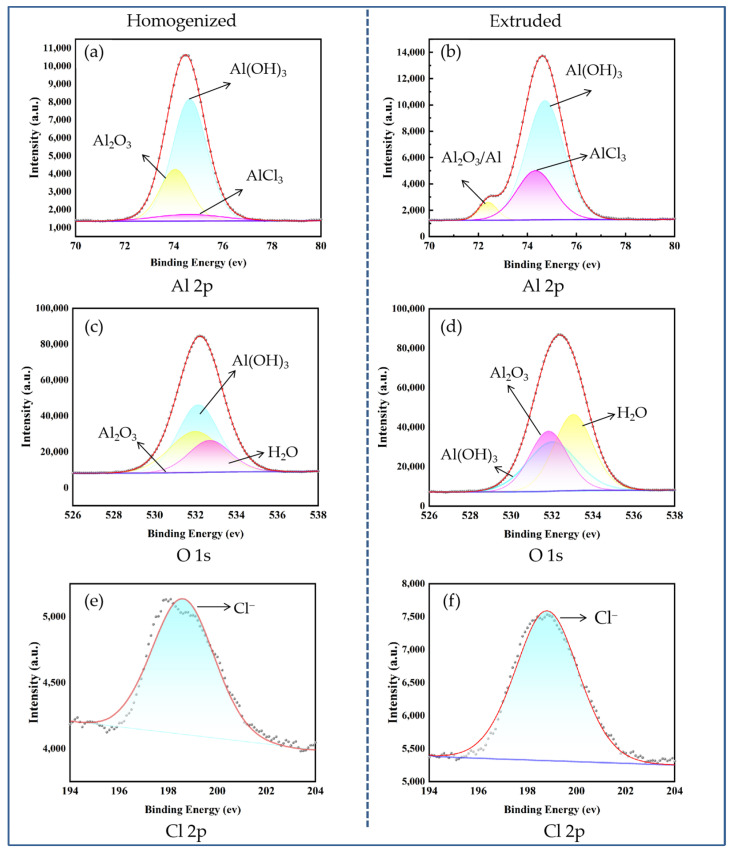
XPS spectra of Al 2p, O 1s, and Cl 2p for corrosion products formed on the homogenized (**a**,**c**,**e**) and extruded (**b**,**d**,**f**) 1100 aluminum alloys after 96 h of acidic salt spray corrosion.

In order to determine the composition of the corrosion products, surface analysis was performed with XPS. Figure 7 shows the XPS analysis of Al 2p, O 1s, and Cl 2p for corrosion products on the surfaces of the homogenized and extruded 1100 aluminum alloys after 96 h of acidic salt spray corrosion. Figure 7a,b exhibit the XPS spectra of Al 2p on the surfaces of the homogenized and extruded 1100 aluminum alloys, respectively, both having peaks at about 75 eV (relative to C 1s = 284.6 eV), corresponding to the oxidized Al (Al^3+^). Figure 7c,d show the XPS spectra of O 1s for corrosion products, and the peaks at binding energies of 531.8, 532.2, and 533.3 eV correspond to O^2−^, OH^−^, and H_2_O, respectively [24]. Combined with the Al 2p spectra, it is evident that the corrosion product layer consisted mainly of Al(OH)_3_ and Al_2_O_3_. However, the difference is that the corrosion products of the homogenized 1100 aluminum alloy were mainly Al(OH)_3_, while the corrosion products of the extruded 1100 aluminum alloy were mainly Al_2_O_3_. Figure 7e,f show the Cl 2p spectra, indicating that the corrosion products of the homogenized and extruded 1100 aluminum alloys both contained a small amount of Cl^−^. The results of XPS are consistent with those of the EDS analysis above, and are similar to the generally accepted aluminum corrosion products of aluminum.

**Figure 8:**
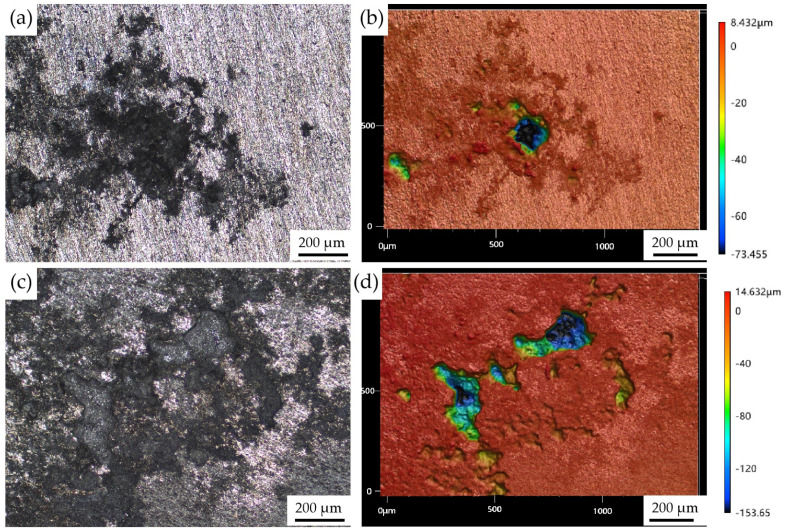
Typical surface morphology of the homogenized (**a**,**b**) and extruded (**c**,**d**) 1100 aluminum alloy after 96 h of acidic salt spray corrosion using laser scanning confocal microscopy.

**Figure 9:**
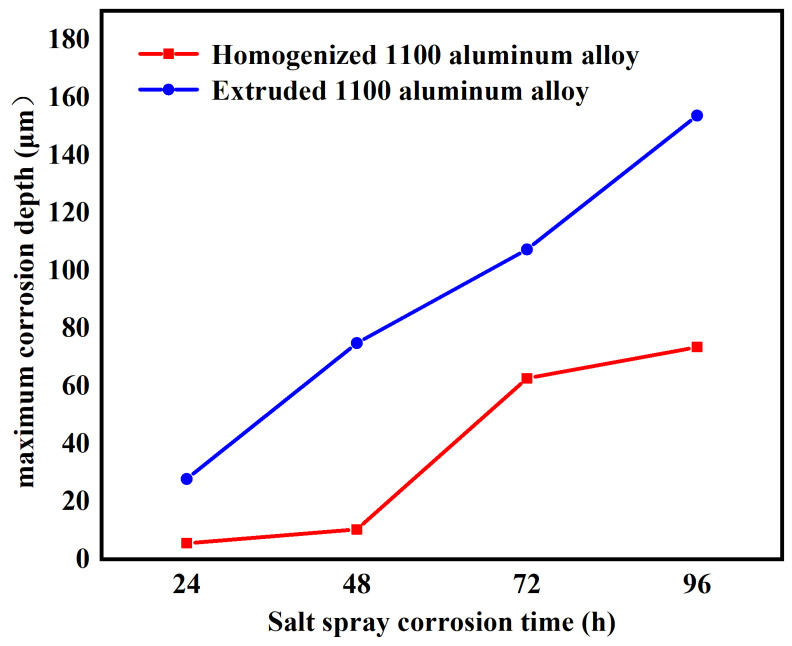
The maximum pitting depth of the homogenized and extruded 1100 aluminum alloy with different salt spray corrosion times.

Confocal microscopy was used to measure the depth and area of the corrosion pit of the homogenized and extruded 1100 aluminum alloy. VK-X3000 multi-file analysis software (https://www.keyence.com/products/microscope/laser-microscope/vk-x3000/) was used for data analysis. Figure 8 shows the localized corrosion morphology of the homogenized and extruded 1100 aluminum alloy after 96 h of acidic salt spray corrosion. Figure 8a,b show the optical images and corresponding surface profiles of the homogenized 1100 aluminum alloy; Figure 8c,d are the optical images and corresponding surface profiles of the extruded 1100 aluminum alloy. It can be seen that the corrosion pits of the extruded 1100 aluminum alloy were larger and deeper than those of the homogeneous 1100 aluminum alloy. Figure 9 shows the maximum pitting depth of the homogenized and extruded 1100 aluminum alloy with different salt spray corrosion times. The maximum pitting depth of the homogenized 1100 aluminum alloy was only 5.51 μm after 24 h of acid salt spray corrosion and 10.3 μm after 48 h of corrosion, and the pitting depth increased slowly. Then, the pitting depth increased rapidly, reaching 62.7 μm after 72 h of corrosion, and subsequently slowed down to reach 73.5 μm after 96 h of corrosion. The maximum pitting depth of the extruded 1100 aluminum alloy was initially 27.8 μm after 24 h of corrosion, then gradually increased with the increase in corrosion time. After 96 h of corrosion, the maximum pitting depth reached 154 μm. The results show that the corrosion rate of the extruded 1100 aluminum alloy in acid salt spray was higher than that of the homogeneous 1100 aluminum alloy, which is consistent with the corrosion surface morphology.

The microstructure analysis shows that the particles of the second phase were coarse in the microstructure of the extruded 1100 aluminum alloy, which affected the perfection of the surface oxide film, and the corrosion began preferably around the particles of the second phase. The extruded 1100 aluminum alloy was more prone to corrosion, which is the reason why the corrosion rate of the extruded 1100 aluminum alloy was fast.

### 3.4. Electrochemical Analysis

**Figure 10:**
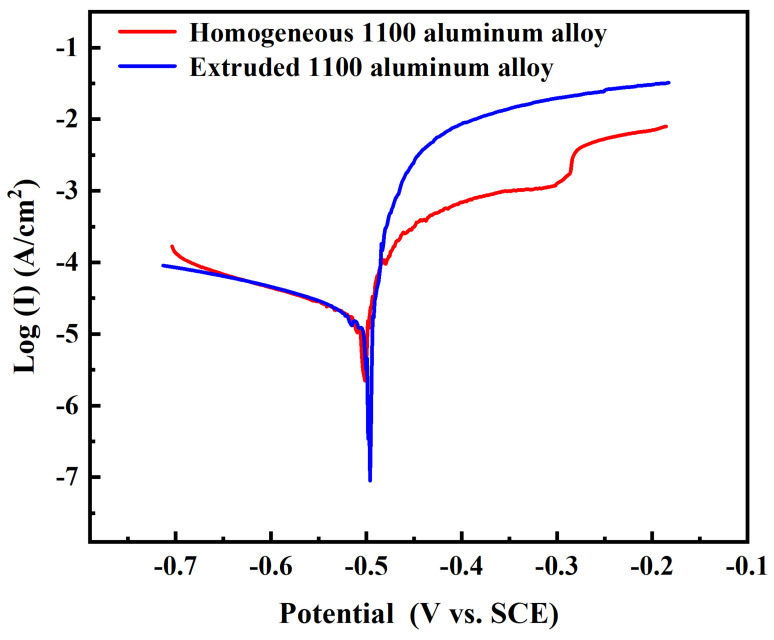
Potentiodynamic polarization curves of homogenized and extruded 1100 aluminum alloy.

Figure 10 shows the potentiodynamic polarization curves of the homogenized and extruded 1100 aluminum alloy measured in acid salt spray corrosion solution. By fitting the polarization curves, the corrosion potential (E_corr_) and corrosion current density (I_corr_) of the 1100 aluminum alloy were calculated, as shown in Table 3. The corrosion potential of the homogenized 1100 aluminum alloy was −0.505 V, and the corrosion current density was 19.3 μA/cm^2^. The corrosion potential of the extruded 1100 aluminum alloy was −0.496 V, and the corrosion current density was 18.0 μA/cm^2^. The corrosion potential of the homogenized 1100 aluminum alloy was slightly lower than that of the extruded aluminum alloy, indicating that the homogenized 1100 aluminum alloy had a higher corrosion tendency. The potentiodynamic polarization curve of the extruded 1100 aluminum alloy had no passivation phenomenon, showing good activation dissolution characteristics.

In the acidic salt spray corrosion solution, the current density of the homogenized 1100 aluminum alloy increased sharply when the potential increased above −505 mV, and the sample was in the active dissolution stage. In the range of −356~−307 mV, the current density basically did not change. At this stage, pitting corrosion slowed down due to the forming of a corrosion product layer on the surface of the aluminum, which slowed the ion diffusion speed. Then, in the range of −307~−278 mV, the current density increased sharply again with the increase in potential, indicating the dissolving of the corrosion product layer. After −278 mV, the characteristic of this interval was that the current density increased slightly with the increase in potential, indicating that the further corrosion products formed on the surface had a protective effect and that the corrosion reaction was weakened.

The corrosion rate can be judged according to the corrosion current density, and the higher the corrosion current density, the higher the corrosion rate [14,25,26]. Under the same corrosion potential, the corrosion current density of the extruded 1100 aluminum alloy was higher than that of the homogenized 1100 aluminum alloy, indicating that the corrosion rate of the extruded 1100 aluminum alloy was fast in the middle and late stages of corrosion, which was also the reason for the significant loss of the extruded 1100 aluminum alloy. This is consistent with the results of the acid salt spray test and the weight loss analysis.

**Figure 11:**
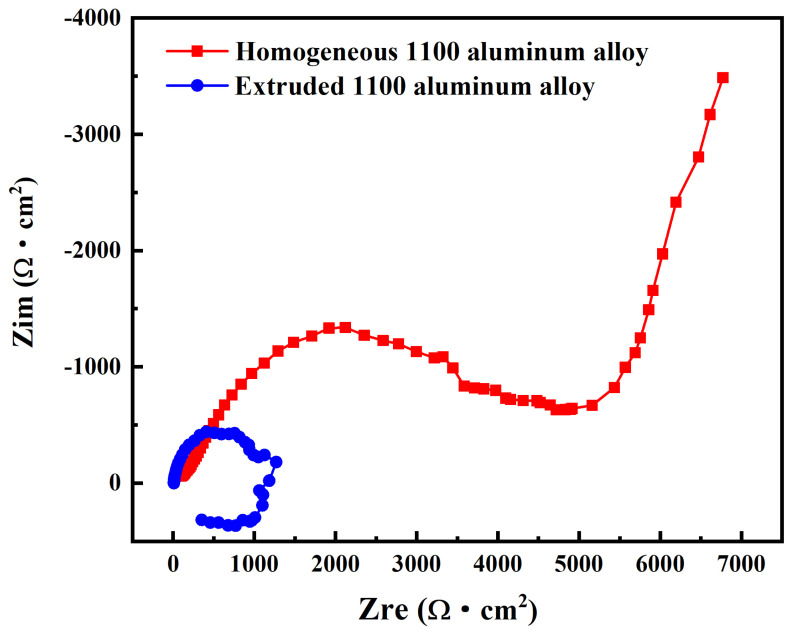
Nyquist diagram of homogenized and extruded 1100 aluminum alloy.

**Figure 12:**
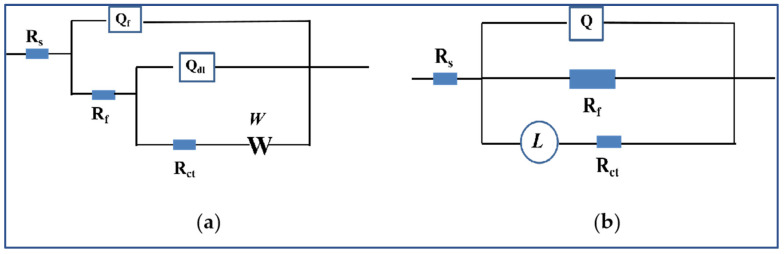
Effective fitting circuit diagram of electrochemical impedance spectra of homogenized (**a**) and extruded (**b**) 1100 aluminum alloys.

Electrochemical impedance spectroscopy (EIS) is an electrochemical measurement method using a small amplitude sinusoidal potential (or current) as a disturbance signal. Figure 11 shows the EIS of the homogenized and extruded 1100 aluminum alloy. It was found that the Nyquist diagram shapes of the two different alloy states were completely different, indicating that the different microstructures led to different corrosion mechanisms. Figure 12 shows the effective fitting circuit diagram of the electrochemical impedance spectrum. R_s_ is the resistance of the bulk solution, R_f_ and Q_f_ are the resistance and constant phase element (CPE) of the oxide film or corrosion product, R_ct_ and Q_dl_ are the charge transfer resistance and constant phase element of the double electric layer, and W is the Warburg impedance. L represents the inductance. The Nyquist diagram of the homogenized 1100 aluminum alloy consists of a capacitance semicircle in the high-frequency region and a diffusion tail in the low-frequency region, while the EIS of the extruded 1100 aluminum alloy in the acid salt spray corrosion solution shows a nearly completely circular induced reactance spectrum. The high-frequency capacitance semicircle represents the charge transfer reaction of the 1100 aluminum alloys with the oxide film, while the low-frequency diffusion impedance is related to the diffusion of oxygen [27]. The diameter of the semicircle in the high-frequency region of the EIS of the homogenized 1100 aluminum alloy is larger than that of the extruded 1100 aluminum alloy, which indicates that the corrosion rate of the homogenized 1100 aluminum alloy was lower than that of the extruded 1100 aluminum alloy under the acid salt spray condition. The EIS of the extruded 1100 aluminum alloy shows a circular inductance impedance, which is due to the absence of a product layer in the corrosion process and the adsorption of intermediate compounds [9,17]. This result is consistent with the morphology of the corrosion products mentioned above.

**Figure 13:**
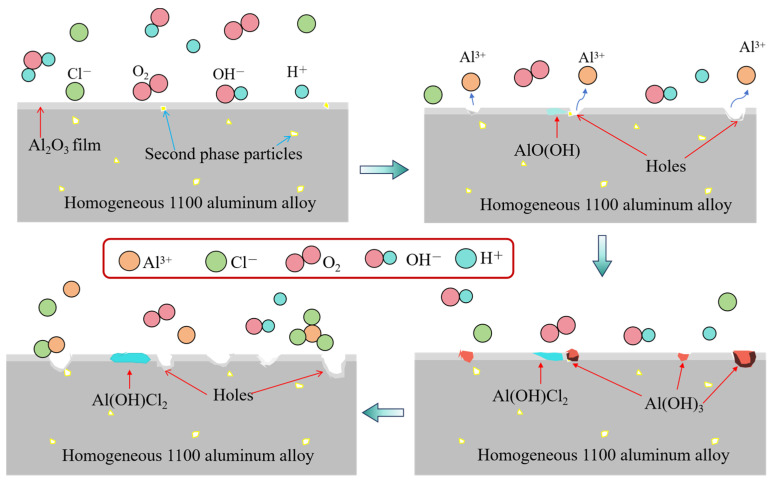
Corrosion reaction mechanism of homogenized 1100 aluminum alloy.

In summary, the corrosion mechanisms of 1100 aluminum alloys in different states are different, and the corrosion mechanisms of homogenized 1100 aluminum alloys in acid salt spray samples are shown in Figure 13. In the acid salt spray test, Al and the trace Al_2_O_3_ protective film on the alloy surface were easily dissolved due to the presence of H^+^ and Cl^−^. An anodic reaction occurred to form Al^3+^.

Al → Al^3+^ + 3e^−^, (2)

Al_2_O_3_ + 6H^+^→2Al^3+^ + 3H_2_O, (3)

According to the electrochemical test results, the corrosion potential of the homogenized 1100 aluminum alloy was lower than that of the extruded 1100 aluminum alloy, indicating that the corrosion dissolution trend of the homogenized 1100 aluminum alloy was higher, and the concentration of Al^3+^ on the surface of the sample increased with the increase in corrosion time. A corrosion product film formed due to the meeting of Al^3+^ ions from the anode reaction with the OH^−^ generated from the cathode reaction to cover the sample surface close to the Al_2_O_3_ particles, and to prevent the dissolution of Al.

O_2_ + H_2_O + 3e^−^ → 4OH^−^, (4)

3OH^−^ + Al^3+^ → Al(OH)_3_↓, (5)

Subsequently, part of Al(OH)_3_ reacted with H_2_O and was converted to Al_2_O_3_·nH_2_O (AlO(OH)). With the increase in corrosion time, more and more corrosion products were formed. Cl^−^ did not easily penetrate into the fresh Al substrates, which led to surface enrichment. Then, Al(OH)_3_ reacted with Cl^−^ to form Al^3+^, forming a corrosion pit.

Al(OH)_3_ + 3Cl^−^ → AlCl_3_ + 3OH^−^, (6)

After the forming of corrosion pits, the corrosion product of Al(OH)_3_ started to form at the periphery of the pits. Meanwhile, the side effects were as follows:

Al^3+^ + Cl^−^ ⇋ AlCl^2+^, (7)

AlCl^2+^ + 2H_2_O → Al(OH)_2_Cl + 2H^+^, (8)

AlCl^2+^ + AlO(OH) → Al(OH)Cl_2_ + AlO^+^, (9)

**Figure 14:**
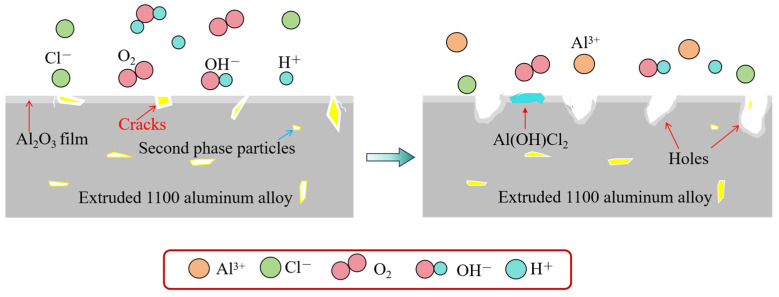
Corrosion reaction mechanism of extruded 1100 aluminum alloy.

The corrosion mechanism of the extruded 1100 aluminum alloy is shown in Figure 14. The corrosion reaction and corrosion products of the extruded 1100 aluminum alloy in salt spray were similar to those of the homogenized aluminum alloy, but the difference is that the corrosion rate of the extruded 1100 aluminum alloy was faster than that of the homogenized aluminum alloy. The corrosion product layer on the extruded 1100 aluminum alloy surface did not play a protective role, and large corrosion pits were directly formed. This is because the microstructure of the aluminum alloy after extrusion was not uniform, with defects around the second phases. The microstructure defects made it easy for Cl^−^ to erode the aluminum matrix. In addition, due to the potential difference between the second phase and the matrix, a large gap around the second phase particles was easily formed through an electrochemical reaction, increasing the corrosion rate and the depth of the corrosion pit. As a result, the corrosion products fell off or dissolved quickly.

## 4. Conclusions

(1) There were second-phase particles containing Al-Fe-Si in the microstructure of the 1100 aluminum alloy, which were network-distributed fine particles in the homogenized microstructure. No dynamic recrystallization occurred during hot extrusion. The microstructure was fibrous, and the second phase was coarse and rod-like.
(2) The surface corrosion morphology of the extruded 1100 aluminum alloy in salt spray was more severe than that of the homogenized 1100 aluminum alloy. The corrosion loss and penetration rate of the homogeneous 1100 aluminum alloy were relatively slow within 48 h, and then rapid corrosion occurred, whereas the initial corrosion rate of the extruded 1100 aluminum alloy was very fast. The results show that the change in microstructure and second-phase particles during hot extrusion accelerated the corrosion rate and deteriorated the corrosion resistance.
(3) With the increase in corrosion time, a layer of corrosion products was formed on the surface of the homogenized 1100 aluminum alloy, which covered the surface of the alloy and slowed the corrosion process. The pitting corrosion of the homogeneous and extruded 1100 aluminum alloys occurred in the initial corrosion stage, but the corrosion rate of the extruded alloy was faster, and deeper corrosion pits were formed.
(4) The corrosion mechanisms of different forms of 1100 aluminum alloys in acid salt spray conditions were different. The corrosion products formed in the initial corrosion stage of the homogeneous 1100 aluminum alloy protected the Al matrix and inhibited the corrosion reaction. With the increase in the corrosion time, the corrosion products dissolved and fell off, an obvious pitting phenomenon occurred, and then the corrosion rate became faster. However, in the initial corrosion stage of the extruded 1100 aluminum alloy, pitting occurred, and the number and depth of corrosion pitting pits increased with the increase in the corrosion time.
(5) In this study, the corrosion behavior of the 1100 aluminum alloy in homogenized and hot-extruded states was compared through an acid salt spray test. The results are expected to help to evaluate the corrosion resistance of the 1100 aluminum alloy in acidic environments, such as in marine engineering, the chemical industry, acid rain, etc.

## Figures and Tables

**Table 1:** Chemical compositions of 1100 aluminum alloy (wt.%).

Specimen	Fe	Si	Cu	Mn	Al
1100 aluminum alloy	0.28	0.09	0.13	0.01	Balance

**Table 2:** EDS results for corrosion products in selected areas in Figure 6.

Specimen	Points	Al		O		Cl	
		wt.%	at.%	wt.%	at.%	wt.%	at.%
Homogenized 1100 aluminum alloy	1	57.59	44.61	42.41	55.39	-	-
	2	37.46	27.25	56.62	69.47	5.92	3.28
	3	27.51	21.17	51.03	66.25	21.47	12.58
	4	44.47	36.07	39.53	54.06	16.00	9.87
Hot-extruded 1100 aluminum alloy	5	74.14	62.96	25.86	37.04	-	-
	6	37.34	26.92	58.01	70.53	4.66	2.55
	7	37.23	31.44	36.12	51.43	22.66	17.13
	8	27.24	21.14	49.96	65.39	22.79	13.46

**Table 3:** The polarization curve fitting parameter values of homogenized and extruded 1100 aluminum alloy.

Specimen	E_corr_ (V_SCE_)	I_corr_ (μA/cm^2^)
Homogenized 1100 aluminum alloy	−0.505	19.275
Extruded 1100 aluminum alloy	−0.496	17.766

## Data Availability

Data is contained within the article.
